# In utero and postnatal exposure to *Foeniculum* vulgare and *Linum usitatissimum* seed extracts: modifications of key enzymes involved in epigenetic regulation and estrogen receptors expression in the offspring’s ovaries of NMRI mice

**DOI:** 10.1186/s12906-023-03875-3

**Published:** 2023-02-14

**Authors:** Fahimeh Pourjafari, Massood Ezzatabadipour, Seyed Noureddin Nematollahi-Mahani, Ali Afgar, Tahereh Haghpanah

**Affiliations:** 1 Department of Anatomical Sciences, School of Medicine, Kerman University of Medical Sciences, Kerman, Iran; 2 Research Center for Hydatid Disease in Iran, Kerman University of Medical Sciences, Kerman, Iran; 3 Student Research Committee, School of Medicine, Kerman University of Medical Sciences, Kerman, Iran

**Keywords:** *Foeniculum vulgare*, *Linum usitatissimum*, Epigenetic modification, Estrogen receptor, First Generation, Ovary

## Abstract

**Background:**

Early-life exposure to exogenous estrogens such as phytoestrogens (plant-derived estrogens) could affect later health through epigenetic modifications. *Foeniculum vulgare* (fennel) and *Linum usitatissimum* (flax) are two common medicinal plants with high phytoestrogen content. Considering the developmental epigenetic programming effect of phytoestrogens, the main goal of the present study was to evaluate the perinatal exposure with life-long exposure to hydroalcoholic extracts of both plants on offspring’s ovarian epigenetic changes and estrogen receptors (ESRs) expression level as signaling cascades triggers of phytoestrogens.

**Methods:**

Pregnant mice were randomly divided into control (CTL) that received no treatment and extract-treated groups that received 500 mg/kg/day of fennel (FV) and flaxseed (FX) alone or in combination (FV + FX) during gestation and lactation. At weaning, female offspring exposed to extracts prenatally remained on the maternal-doses diets until puberty. Then, the ovaries were collected for morphometric studies and quantitative real-time PCR analysis.

**Results:**

A reduction in mRNA transcripts of the epigenetic modifying enzymes DNMTs and HDACs as well as estrogen receptors was observed in the FV and FX groups compared to the CTL group. Interestingly, an increase in ESRα/ESRβ ratio along with HDAC2 overexpression was observed in the FV + FX group.

**Conclusion:**

Our findings clearly show a positive relationship between pre and postnatal exposure to fennel and flaxseed extracts, ovarian epigenetic changes, and estrogen receptors expression, which may affect the estrogen signaling pathway. However, due to the high phytoestrogen contents of these extracts, the use of these plants in humans requires more detailed investigations.

## Background

The ovary is the main organ of female reproductive system. In utero development of this organ highly depends on the signaling pathways of endogenous estrogen hormone, hence developmental exposure to exogenous estrogens may disturb this process, and affect offspring’s endocrine system and ovarian function [1].

One of the main important group of the environmentally exogenous estrogens is phytoestrogens; dietary estrogens produced by plants that are structurally or functionally similar to endogenous estrogens. These substances are divided into four groups: lignan, stilbenz, isoflavonoids, and flavonoids [2]. They work by activating estrogen receptors in a variety of organs, including the ovary and uterus, to have effects that are comparable to those of endogenous estrogen [3]. Depending on the cell type, dose and time of exposure, they can have estrogenic or antiestrogenic effects [4]. The exposure effects of different phytoestrogens on the ovarian function, apoptosis and estrogen receptors (ESRs) expression of the female reproductive system were determined [5–7]. Besides, regarding to the placental and milk transmission of phytoestrogens, these bioactive compounds might affect the later health status of the offspring. Various effects such as premature puberty, impaired ovarian folliculogenesis and estrous cycle as well as impaired anogenital distance were reported following prenatal and postnatal exposure to phytoestrogens [8, 9].

Among the medicinal plants, *foeniculum vulgare*, commonly known as fennel, and *Linum usitatissimum,* commonly known as flax, are prevalent plants in the Mediterranean region. They used in traditional medicine to woman’s health and treat a variety of diseases [10–12]. Therefore, these plants are traditionally used to make Qavut in Iran (Kerman provinence) and fennel could increase breast milk production [13]. They are rich in phytoestrogens, as fennel contains a high concentration of phytoestrogenic compounds such as flavonoids included of eriodictyol, quercetin-3-rotinoside, and rosmarinic acid [14], and flax has highest level of lignans [15] among different medicinal plants. The positive effects of fennel and flaxseed exposure on ovarian function have yet been well determined [16–18]. Furthermore, flax root lignans could alter the expression of ESRs in the breast cancer cells [19]. We have recently reported that pre and postnatal exposure of mice to hydroalcoholic extracts of both plants could alter the ovarian folliculogenesis, apoptotic genes expression and oxidative stress in first generation (F1) of female offspring [20–22].

However, maternal lifestyle factors like diet [23, 24], especially during the time of development, may change how genes are expressed via epigenetic phenomena. Because these changes are independent of genomic sequencing alteration, they may have an impact on future health or disease risk [25, 26]. Three mechanisms of the epigenetic modifications are DNA methylation, histone modification, and regulation of non-coding RNAs. DNA methylation is performed by the DNA methyltransferases (DNMTs); DNMT1, DNMT3A, and DNMT3B. Also, histone modifications are done by histone acetyltransferase (HAT) and deacetylases (HDACs) enzymes. A previous study showed that pre and postnatal exposure to a phytoestrogen-rich diet containing genistein and daidzein can alter DNA methylation of the ESRα gene in the female offspring [27]. Such alterations could have long-term effects to prevent the ovarian and prostate cancers by modulating DNMTs and HDACs activities [28].

However, the overall safety, hazardous and beneficial levels of a dietary regimen supplemented with plants that are high in phytoestrogens on the long-term health of the offspring are still unknown, despite recent increases in research on the effects of phytoestrogens on health status. Therefore, regarding the potential of phytoestrogens in epigenetic alterations, for the first time, we aimed to examine the early-life exposure to fennel and flaxseed-based diets, which are very rich in natural phytoestrogens, on the expression level of enzymes involved in the epigenetic modifications of the ovaries in adulthood.

Despite the similar biological activity of phytoestrogens as the endogenous estrogen on ESRs and the importance of signaling pathways of ESRs in the differentiation and maintenance of female reproductive tissues, so far, no study has surveyed the effect of perinatal exposure with lifelong exposure to hydroalcoholic extracts of these plants on the expression level of the ovarian ESRs in adulthood, that’s what we evaluated in the current study.

## Methods

### Preparation of hydroalcoholic extracts

Fennel and flaxseed were prepared from market in Kerman, Iran, were kindly confirmed by Dr. Fariba Sharififar and a voucher specimen of fennel (Voucher number: KF1466) and flaxseed (Voucher number: KF1628) were deposited at the Herbarium Center at the Department of Pharmacognosy, Kerman University of Medical Sciences, Kerman, Iran. Experimental research and field studies on plants including the collection of plant material are complying with relevant guidelines and regulation. The extracts were prepared by using warm maceration method. Briefly, dried seeds were milled and passed through a sieve (mesh 300). Then, 100 g powdered seeds were soaked in ethanol solution 80% for 72 h. The obtained extracts were then concentrated in vacuo and dried in an oven at 40 °C for 48 h. The extract was kept at -20 °C until use. Considering that flavonoids are the most of the compounds of fennel and flax, therefore, in order to standardize the plant extracts, thin layer chromatography was carried out and the predominant flavonoid of the plant was determined in our previous study [22].

### Animals and study protocol

Sixteen female and eight male naval medical research institute (NMRI) mice weighting 25–30 g (6–8 weeks old) were obtained from the animal house of Afzalipour School of Medicine (Kerman, Iran). The animals were treated in compliance with the guidelines for the care and use of animals approved by the institutional ethics committee of Kerman Medical University (approval number: IR.KMU.REC.1400.472). The animals were kept in cages covering wood fiber bedding (4 mice/ cage) in well-controlled conditions of temperature (21 ± 2 °C) and 12/12 h of light/dark cycle with free access to water and standard rodent diet.

**Fig. 1:**
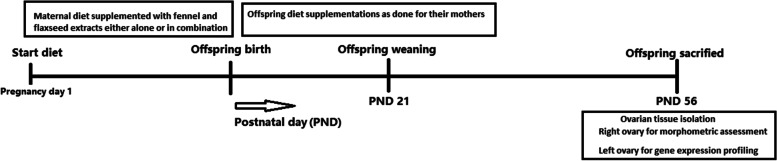
Experimental plan. By observing the vaginal plug at pregnancy day 1, the pregnant mice randomly fed fennel and flaxseed extracts, either alone or in combination, during pregnancy and lactation periods. After offspring weaning at postnatal day 21 (PND 21), they continue their mother’s diet until PND 56. At this day, the offspring sacrificed for assessment of ovarian tissue morphometry and gene expression profiling

One male and two female mice were placed in each cage for mating. The next day, female mice were evaluated for vaginal plaque formation that was identified as the gestation day 0 (GD 0). As shown in Fig. 1, pregnant mice (*n* = 32) were randomly assigned to following experimental groups: control group (CTL), in which the animals consumed a standard rodent chow; FV group, which the animals received hydroalcoholic extract of fennel (500 mg/kg/day) [20]; FX group, which the animals received hydroalcoholic extract of flaxseed (500 mg/kg/day) [20]; and FX + FV group, which animals received hydroalcoholic extract of flax (500 mg/kg/day) and fennel seed (500 mg/kg/day). Based on the our previous studies [20, 22], a dose of 500 mg/Kg was chosen for both extracts as an effective dose. In order to intake a daily dose of 500 mg/Kg of extract by animals, first, in a pilot study, the required amount of extract was measured based on the animal weight for one week. Also, the food consumed by the animal was also evaluated weekly. Then, this amount of extract was mixed with weekly desired animal standard diet and was given to the animal for one week. Considering the different daily food intake of pregnant and lactating mice, new calculations were also made for their consumption. It should be mentioned that all the mice in one cage were in the same stage of development. These extracts were added to the animal’s diet from the first day of pregnancy, continued during lactation period, after weaning, the pups received the same treatment as mothers did until 56th postnatal day (PND). we choose continuous treatment from offspring weaning until the onset of puberty (PND 56) due to that different specific organs such as female reproductive system is not completely differentiated at birth, but continues to experience cellular differentiation until just prior to the onset of puberty [29].

After this period, female pups were euthanized by ketamine (5–10 mg/kg) and xylazine (5 mg/kg) (the combination of ketamine as a dissociative agent and xylazine as a 2- adrenoreceptor agonist is one of the firmly-established overall anesthetic modalities usually used for laboratory mice). The total number of used pups was 32 animals (*n* = 8/group) that the pups in each group were arise from eight pregnant mice. After a wide literature review and available methods such as E and Power analysis methods, this sample size was set. There were no excluded animals during treatments.

### Determination of ovarian morphology

At post-natal day 56 (PND56) between 8–10 am, the female mice were weighted by a digital scale (Sartoroius, Japan) and euthanized by a mixture of ketamine and xylazine. Right ovary was carefully removed, weighted and its large and small diameters were measured using a digital caliper)Mitsubishi, Japan). Ovarian coefficient was calculated by dividing the ovary weight by the mouse weight* 100 [30].

### Quantitative reverse transcriptase–polymerase chain reaction (qRT-PCR)

qRT-PCR was performed on the left ovaries to assess the gene expression levels of enzymes involved in epigenetics modifications: DNMT1, DNMT3A, DNMT3B, HDAC1, HDAC2 as well as estrogen receptor α and β. Total RNA was isolated after addition of TRIzol reagent (CINNAGEN, Iran). The quantity and quality of RNA were determined using NanoDrop 2000 spectrophotometer (Thermo fisher scientific, Wilmington, DE, United States) and electrophoresis, respectively. Complementary DNA (cDNA) synthesis from total RNA (1 μg) was performed according to the manufacturer's protocol (cat.no.YT4500, YTA) by a thermocycler (BIOMETRA, Germany). The synthesized cDNA was stored at -20 °C until use. Three replicates of qRT-PCR reactions were performed on a mixture consisted of 5 μl syber green Master Mix (Genaxxon bioscience, Ulm, Germany), 1 μl of each specific primers [23] (Table 1), and 2 μl of cDNA in a final volume of 10 μl. The program of q real-time PCR was as follows: initial denaturation at 95 °C for 40 s, followed by 35 cycles of denaturation at 95 °C for 20 s, 60 °C for 30 s, and final extension at 72 °C for 30 s using a Light Cycler Real-Time PCR System (MIC, Queensland, Australia). Beta actin was assigned as the reference gene. All qPCR data were subsequently analyzed using the 2^_ΔΔCT^ method [23].

**Table 1:** Primers used for real-time RT-PCR

Genes	Primer sequence (5^´^-3^´^ orientation)	Annealing temp (°C)	Product size (bp)	Gene bank accession no
*DNMT3a*	F: TGATGGGATTGCTACAGGGCT	62		
	R: ATGCTTCTGTGTGACGCTGC		163	NM_007872.4
*DNMT3b*	F: CCAGCCTCACGACAGGAAACA	62	78	NM_001271747.1
	R: CTCCTCATACCCGCTGGCAC			
*DNMT1*	F:GACAGTGACACCCTTTCAGTTG	64	94	NM_001199431.2
	R: CCTTCGTGAAGTGAGCCGTG			
*HDAC1*	F: ACGGCATTGACGACGAATCCT	64	158	NM_008228.3
	R: GCGTGTCCTTTGATGGTCAGA			
*HDAC2*	F: ACGGGTGGTTCAGTTGCTGG	64	77	NM_008229.2
	R: AGTCCTCCAGCCCAATTGACAG			
*Estrogen receptor 1 (alpha):ESR1*	F: ATTCTTCTCAAGCAGGTGGCCC			
	R: GCTCCAGCTCGTTCCCTTGG	61	159	NM_007956.5
*Estrogen receptor 2 (beta): ESR2*	F: GAGTGCTGTCCCAAGGGATGA			
	R: CGCCAGGAGCATGTCAAAGAT	57	70	NM_207707.1
*-βactin*	F: GTCCACACCCGCCACCAGTT		65	NM_007393.5
	R: GAGCCGTTGTCGACGACCAG	60		

### Statistical analysis

Statistical analysis of data was done by Statistical Package for the Social Sciences (SPSS software version 16) (https:// spss. software. informer. com/ 16.0/). First, the normal or abnormal distributions of data were investigated using One-sample Kolmogorov–Smirnov test. If the parameters had a normal distribution (morphometric data), the one-way ANOVA test followed by post hoc *Tukey* was used and otherwise (gene expression), the non-parametric Kruskal–Wallis test was used. The GraphPad Prism, version 6 (GraphPad Sofware, San Diego, CA; (https:// getpc soft. wikis end. com/ tips/ Graph pad_ Prism_6_ Full_ Versi on_ Free_ Downl oad. html) as used for graphing. Data were expressed as Mean ± standard error of the mean (SEM). *P* ≤ 0.05 was considered statistically significant. The outcome assessment and also data analysis was done in the blind form.

## Results

### The effect of pre- and postnatal exposure to a diet regimen supplemented with fennel and flaxseed extract on mice weight, ovarian weight, coefficient and diameters

As shown in Table 2, body weight of pups exposed to fennel was significantly higher than the control and FX groups (CTL; *P* = 0.014, FX; *P* = 0.013, respectively). Also a significant (*P* = 0.024) increase in the ovary weight and coefficient of the FV group was detected in comparison with that of the exposed to FX. Also, a marked increase in the large diameter of the ovaries in the FX + FV group was observed compared to the CTL and FX groups (compared to the CTL; *P* = 0.032, compared to the FX; *P* = 0.014) (Table 2).

**Table 2:** The effect of pre- and postnatal exposure to a diet supplemented with fennel and flaxseed extracts on mice body weight and ovarian morphometric indexes

*Variables*	*Groups*			
	**CTL**	**Flax** **(FX)**	**Fennel** **(FV)**	**Flax + Fennel** **(FX + FV)**
Body weight (g)	23.21 ± 0.86	23.2 ± 0.53	25.68 ± 0.31^a*b*^	24.55 ± 0.5
Ovary weight (mg)	3.7 ± 0.5	3 ± 0.6	5.5 ± 0.68^b*^	5.1 ± 0.5
Ovary/ body weight (mg/g)	0.016 ± 0.002	0.013 ± 0.003	0.0217 ± 0.003^b*^	0.0208 ± 0.001
Large ovary diameter (mm)	2.12 ± 0.11	2.04 ± 0.22	2.6 ± 0.16	2.79 ± 0.10^a*b*^
Small ovary diameter (mm)	1.43 ± 0.07	1.27 ± 0.12	1.61 ± 0.12	1.5 ± 0.03

^a^ shows a significant difference compared to the control group and ^b^ shows a significant difference compared to the flax-received mice. * shows *P*< 0.05

### The effect of pre- and postnatal exposure to a diet regimen supplemented with fennel and flaxseed extract on mRNA expression of major enzymes involved in epigenetic modification

#### Expression levels of DNA* methyltransferases*

**Fig. 2:**
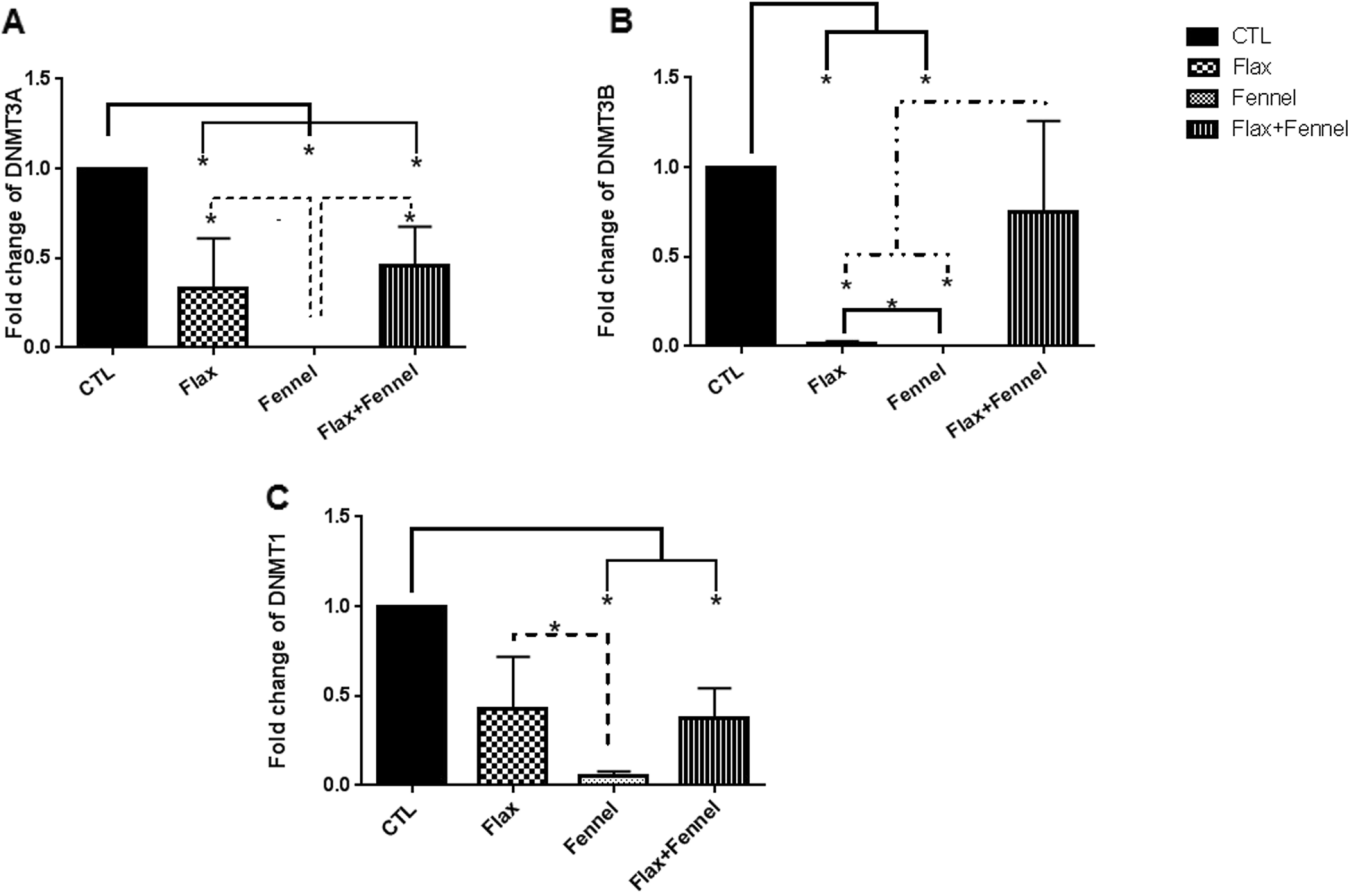
DNMTs expression level in the F1 ovarian tissue after exposure to the fennel and flaxseed extracts. The expression level of DNMTs (DNMT3A, 3B and DNMT1) decreased in all ovaries of treated mice compared with the control group. The mRNA level of each sample was normalized against β actin mRNA level. Data are presented as mean ± SEM. * shows *P* ≤ 0.05. Kruskal–Wallis test was used for DNMTs expression level analysis. The authors performed three replications

Analysis of real-time PCR data revealed that the mRNA expression level of DNMT3A significantly decreased in all groups when compared to that of the control group (*P* = 0.03). However, the lowest level of this transcript was observed in the FV group with a significant difference with the FX and FX + FV groups (*P* = 0.05) (Fig. 2A).

Furthermore, as shown in Fig. 2B, a significant (*P* < 0.05) decrease in the expression of DNMT3B was observed in all treated groups compared to that of the CTL group, however this difference reached to a significant level in the ovarian tissues of pups that pre- and postnatal exposed to a diet regimen supplemented with flax and also fennel, alone (*P* = 0.03), although this value was significantly lower in the FV group compared to the FX group. Also, a statistically significant difference (*P* = 0.05) was observed between the FX and FV groups, alone as well as compared to the FV + FX pups.

Moreover, expression of DNMT1, decreased in all treated groups, with a significant (*P* = 0.03) difference with that of the FV and FX + FV groups (Fig. 2C). Also, a significant difference was observed between the flax and fennel groups (*P* = 0.05) based on this value (Fig. 2C).

#### Expression levels of Histone deacetylates (H*DACs)*

**Fig. 3:**
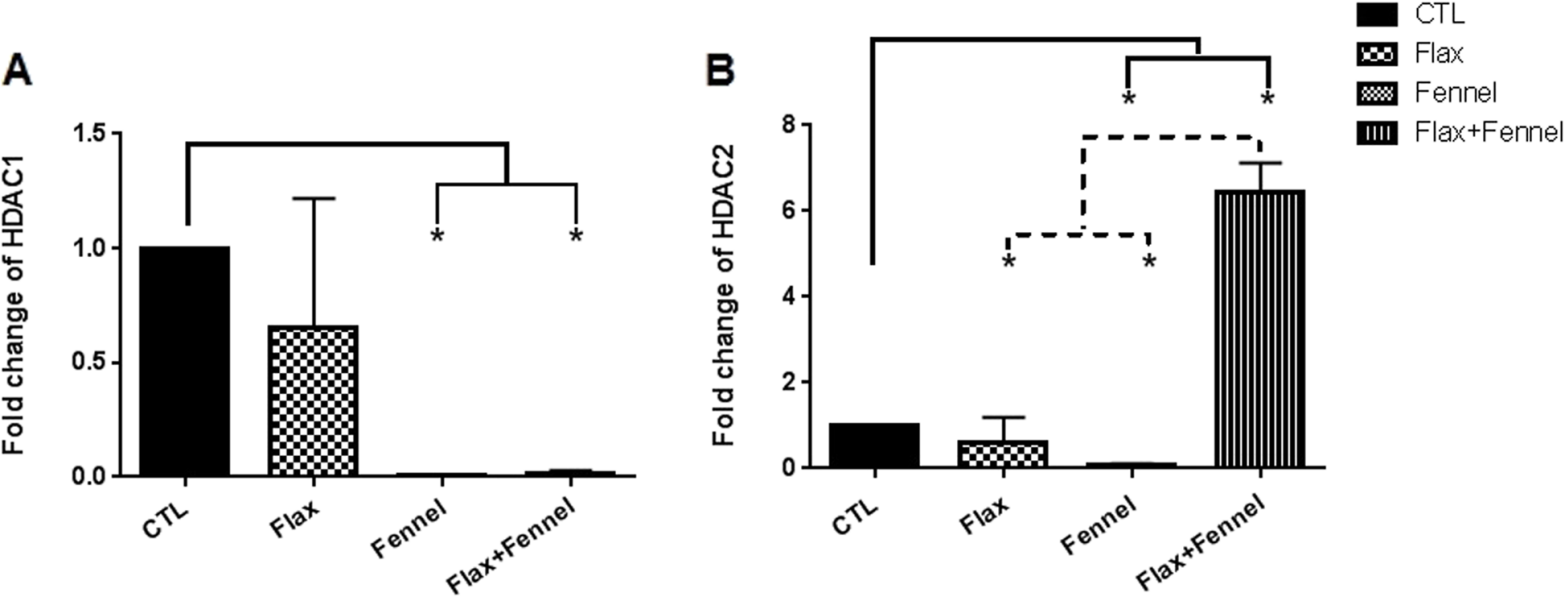
HDACs expression level in the F1 ovarian tissue after exposure to the fennel and flaxseed extracts. The expression level of HDAC1 decreased significantly in the ovaries of fennel and also flax + fennel-treated mice compared with the control group. Based on the HDAC2 expression level, a marked reduction was observed in the fennel-exposed mice, while in the concomitantly-exposed mice, a sharp raise was observed. The mRNA level of each sample was normalized against β- actin mRNA level. Data are presented as mean ± SEM. * shows *P* ≤ 0.05. Kruskal–Wallis test was used for HDACs expression level analysis. The authors performed three replications

In the current study, the mRNA expression of enzymes involved in the histone modifications including HDAC1 and HDAC2 was evaluated. As shown in Fig. 3, qRT-PCR data showed that the expression level of HDAC1 decreased in all exposed offspring, with a significant reduction in the FV and FX + FV groups compared with the CTL group (*P* = 0.03) (Fig. 3A).

Moreover, the expression level of HDAC2, similar to HDAC1 transcripts, reduced in the FX and FV groups with a significant (*P* = 0.03) value of the FV group compared with the CTL group. In contrast, a marked increase in mRNA level of HDAC2 was observed in the FV + FX group compared to those of the CTL (*P* = 0.03), FX (*P* = 0.04) and FV (*P* = 0.04) groups.

### The effect of pre- and postnatal exposure to a diet regimen supplemented with fennel and flaxseed extracts on mRNA expression of estrogen receptors

**Fig. 4:**
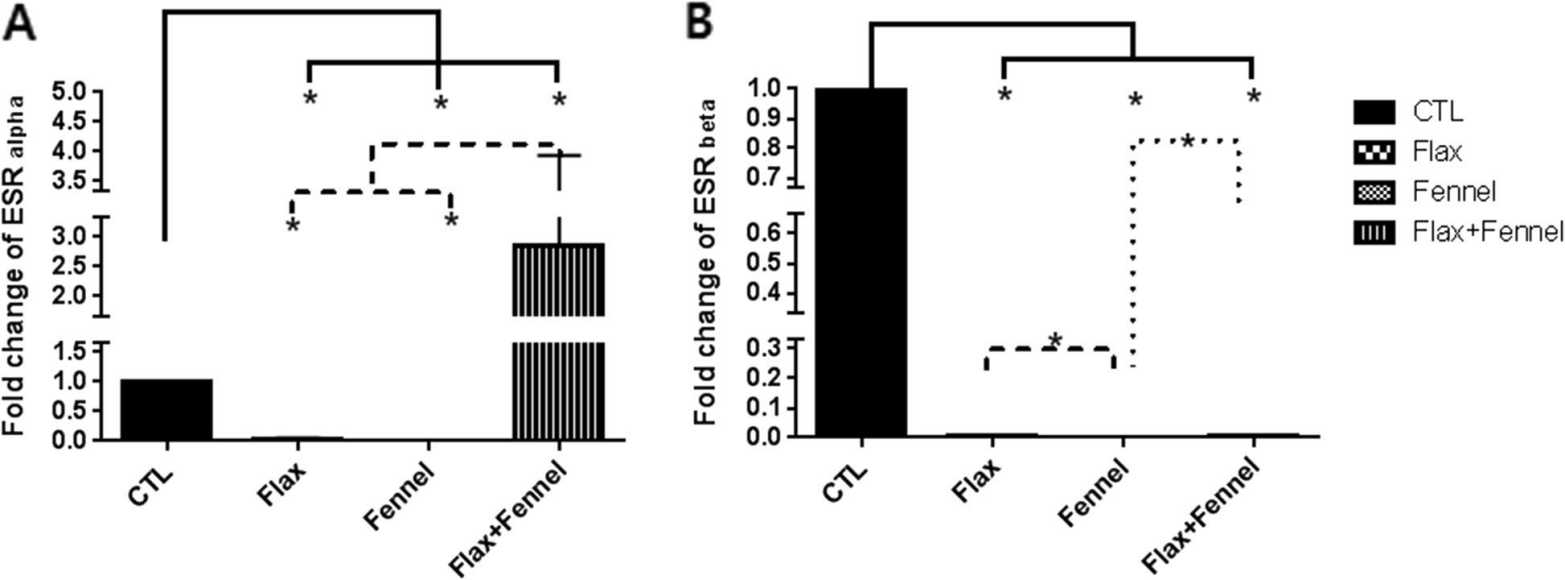
Estrogen receptors expression level in the F1 ovarian tissue after exposure to the fennel and flaxseed extracts. Relative expression levels of both isoforms of estrogen receptors included of ESRα and ESRβ in the left ovarian of adult mice after treatment with hydroalcoholic extract of fennel and flax seed, either alone or in combination showed a marked decrease in the expression level of both ESRs, although the expression level of ESR alpha increased significantly in the concomitantly-exposed mice. The mRNA level of each sample was normalized against β actin mRNA level. Data are presented as mean ± SEM. * shows *p* ≤ 0.05. One-Way ANOVA test followed by Tukey *post-hoc* was carried out for ESR alpha expression level analysis and Kruskal–Wallis test was used for ESR beta. The authors performed three replications

qRT-PCR data revealed a significant reduction in the expression levels of ESRα and ESRβ in the FX (*P* = 0.03) and FV groups (*P* = 0.03) when compared to that of the CRT group. In contrast, ESRα gene expression in the FX + FV group significantly (*P* = 0.03) increased while the expression of ESRβ significantly (*P* = 0.03) decreased compared with the CTL group (Fig. 4).

## Discussion

This in vivo study is the first to reveal that prenatal and postnatal exposure to the fennel and flaxseed extracts could lead to F1 ovarian epigenetic changes via differential expression of DNMTs and HDACs as well as ESRs. These changes might affect the normal function of the ovary in adulthood.

Complex molecular regulations during in utero development are a result of interactions between genetic and epigenetic. Maternal lifestyle [23, 24], such as a dietary supplement with phytoestrogens is one of the environmentally epigenetic effect elements that could affect developmental programming processes [26]. However, maternal steroid hormone-binding protein (SHBG) and fetal α-fetoprotein are the main modifiers of fetal exposure to phytoestrogens that may protect the fetus partly from the destructive effects of exogenous estrogens such as phytoestrogens [31]. Besides, breast milk could be another transporter pathway for infants' phytoestrogen exposure. Because the reproductive system and germ cells are developing at these critical times, phytoestrogens may act as exogenous estrogens, making them particularly vulnerable. To date, several studies extensively determined [1] that early developmental exposure to environmental estrogens could reduce follicular growth and primordial follicle numbers and alter follicle assembly, resulting in the formation of ovarian cysts and multi-oocyte follicles [31], therefore would lead to lower concise reproductive lifespan. Nevertheless, although there is limited information on ovarian epigenetic changes after individual exposure [32], to date, there is no evidence regarding ovarian epigenome alteration following long-term exposure to phytoestrogens.

One of the main underlying mechanisms involved in epigenetic modifications is the alteration of the DNA methylation status of specific genes. In general, DNA methylation was occurring by two types of DNMT; de novo (DNMT 3A and 3B) and maintenance (DNMT1) [33]. Our data showed an overall decrease of DNMTs expression in the ovarian tissue of all exposed F1 mice, especially in the fennel exposed mice. Recently, Harlid et al. [34] reported that neonatal exposure to soy’s phytoestrogens in the formula could lead to alteration of DNA methylation level in vaginal epithelial cells, which may be related with reduced estrogen-responsive genes and probably later-life health problems. Besides, phytoestrogens are sometimes referred to as endocrine disruptors. There are several reports that early life exposure to several endocrine disruptors with hormone-like activity, such as methoxychlor (MXC) [35], bisphenol A (BPA) [36], and diethylhexyl phthalate (DEHP) [37] could change ovarian DNA methylation. Abnormal methylation is potentially related to differential individual vulnerability to the later development of disease, such as various cancers [38, 39]. It was shown that overexpression of DNMTs could cause hypermethylation of tumor suppressor genes resulting in cell transformation in different cancers [40, 41]. In addition to DNA methylation, histone aberrant acetylation pattern is also associated with the modification of epigenetic signature. HDACs family members are involved in the suppression of DNA transcription by removing the acetyl group from histones. A raise in HDACs was reported in cell proliferation and invasion through repression of tumor suppressor genes [42, 43]. To date, 18 different mammalian HDACs have been categorized into four classes [44, 45]. Class I including HDAC 1, 2, 3, and 8 are mostly involved in carcinogenesis compared to others [46]. It has been revealed that there was a decrease of DNMTs (DNMT1, DNMT 3A, and DNMT3B) as well as HDAC2 expression levels in the mammary gland of F1 rats that underwent a phytoestrogen-enriched regimen during in utero development, breastfeeding, and puberty periods [47]. Also, one previous study reported decreased expression levels of DNMT1, DNMT3A, DNMT3B as well as HDAC1 and HDAC2 in the uterus of adult offspring following neonatal genistein exposure [48]. In the present study, the downregulations of HDAC1 and HDAC2 were observed in the ovary of adult mice that were exposed to either fennel or flaxseed extracts. It is well established that HDACs have a physiological role in cell proliferation and survival, so that overexpression of HDACs I and II has been seen in the various tumor cells [49], including ovarian cancer tissue [50]. Hence, HDAC inhibitors have been used as tumor therapeutic agents by inducing apoptosis [51]. Nowadays, there is special attention to phytoestrogens for cancer therapy because of their effects on reducing the HDACs expression and also activity [52, 53], although some reports reported no marked effects [54]. Different used doses and durations may explain the contradictory results [55]. Our findings suggest that each of these plants may function as an HDAC expression down-regulator in the ovary, and as a result, they may be considered an element that protects the ovary by preventing abnormal cell growth and cell cycle progression, likely through hypomethylation and histone deacetylation of tumor suppressor genes. A decreased growth of renal cancer cell lines via decreasing of DNMTs and HDACs activity was seen following exposure to the genistein phytoestrogen in a dose-dependently manner [56]. However, similar to DNMTs, reduction of HDACs transcriptions was pronounced in the pups that were exposed to the fennel compared to the flaxseed. Hence, ovarian epigenome alteration was more affected by fennel rather than flaxseed.

On the one hand, one of the target sites for these epigenetic enzymes' function is the promoter region of the estrogen receptors; ESRα and ESRβ. Both isoforms are involved in the ovarian development, by triggering the estrogen signaling pathway. Similar to the endogenous 17-beta estradiol, and competitively affinity, phytoestrogens act on these receptors, although ESRβ is a selective receptor for them, therefore exposure to these plant-derived estrogenic compounds could affect reproductive biology, development and physiology. It was shown that early-life exposure to several chemicals with hormone-like activity results in long-term changes in the ESRβ gene in the ovary. Altered ESRs activity might lead to changed ovarian folliculogenesis and steroidogenesis. However, different ESRs expressions could be because of the modification of the epigenetic mechanisms that regulate it. DNA methylation of promotor regions of genes by DNMTs, which usually results in gene expression suppression, is one of them. Several reports showed endocrine disruptors could lead to high expression levels of DNMTs in the ovary. Changes in ovarian DNMT expression can disrupt the expression level of many transcription factors, which regulate the gene expression associated with the hormone action, paracrine growth factors, and extrinsic innervation. For example, MXC could induce DNMT and hypermethylation of ESRβ promoter region in the ovary as a result lead to a decrease of ESRβ protein, lower response to endogenous circulating estradiol (E2) and the alteration of E2-dependent follicular development in adulthood. Therefore, a relationship between decreased ESRβ expression and increased DNMT expression was determined. However, as yet, such a relationship was not studied in the adult ovary following long-term exposure to phytoestrogens, which is evaluated in the current study. Our data for the first showed a differential expression of ESRs, along with the epigenetic changes in one direction, in the ovarian tissues of all pups that were exposed to fennel and flaxseed. Nevertheless, evaluating of the methylation level of ESR promoters along with the changes in the expression level of epigenetic modification enzymes may provide more complementary information. Previous studies [35, 57] reported that pre and neonatal exposure to some endocrine disruptors could cause ovarian dysfunction via a reduction of ESRβ expression because of DNMT3B overexpression and hypermethylation of the ESRβ promoter region that finally leads to a lesser response to endogenous estrogen.

However, regarding the reduced expression level of both ESRs, fennel and flax, alone may act as agonists leading to ESRs down-regulation following their long-term exposure. This decrease could cause a lower response to E2 in adulthood and change the folliculogenesis regulated by E2 and several paracrine factors such as anti- mullerian hormone (AMH). As reported in our previous study [22], the serum estradiol level was highest in the fennel exposed mice and lowest in the flax-received mice, while animals that received the fennel and flax seed concomitantly had a lower level rather than fennel. An invert relationship [58], between circulating E2 level and ESRs mRNA Level was reported as ovariectomized animals had a lowest level of E2 along with the increasing ESRs mRNA and treatment with E2 led to increasing circulating E2 along with decrease of ESRs expression level. In agreement with them, we found a high level of circulating E2 in the fennel group while exhibited a sharp reduction of ESRs expression. The flaxseed-exposed mice with low circulating E2 had higher level of ESRs mRNA in compared to the fennel and flax + fennel-exposed mice. Besides, in the combined group, the circulating E2 was less than fennel group and ESR beta mRNA was higher than fennel-exposed mice and ESR alpha was very greater than fennel and flaxseed-exposed mice.

It is well showed that the function of these isoforms of ESRs is different so that almost, anti-proliferative effects are related to the ERβ and proliferative effects are related to the ERα. Ultimately, the ratio of ESRα-to β could determine the net effect of these plant-derived compounds on the target tissue. A high ratio may associate with cellular proliferation stimulation, while the opposite effect was seen at a low ratio. A previous study showed that a low dose of biochanin A; a type of dietary phytoestrogens could stimulate the growth of human mammary carcinoma cells by increasing of ESRs expression level while exhibiting the opposite effect at high dose [59]. However, phytoestrogens have the potential to alter ESR conformation, leading to partial agonist and antagonist properties dependent on the target location and their dose. Based on our data, these extracts may be potential candidates for finding herbal chemo-protective drugs via the overall reduction of the ESRα to β ratio, although more research is needed to test this issue. Surprisingly, those animals that were exposed to a mixture of fennel and flaxseed extracts showed a rise in the mRNA expression of ESRα along with ESRβ reduction. Considering that the overall ratio is toward increasing the ESRα/β ratio, therefore exposure to the very high levels of phytoestrogens in a mixture of these plants might lead to re-stimulation of growth and division of cells, which is seen in almost cancers, such as breast cancer. However, we observed overexpression of HDAC2 along with ESRα in the ovaries of pups that were exposed to both extracts, simultaneously, which is in disagreement with some studies that determined the high activity of HDACs are related to estrogen receptor suppression [49]. Regarding increasing of ESRα /β ratio [60] as well as overexpression of HDAC2 [46, 61, 62] that have been reported in several tumor cells, such as ovarian cancer, our data make us more cautious in the long-lasting exposure to the high concentrations of complex phytoestrogens in the combination of these two plants. In line with our data, some animal studies reported that neonatal exposure to genistein could cause an enhanced risk of tumors [63]. However, as yet, there is no conclusive ground regarding the action of phytoestrogens as antitumor or tumor growth stimulation elements [64].

Therefore, our data clearly showed the changes of the ovarian epigenome. This alteration might affect the oocyte development by the alteration of the expression level of genes-involved in the secretion of ovarian metabolite, endocrine and paracrine factors, derived from ovarian somatic cells, that progress the oocyte maturation and leading to transgenerational phenotypes in reproductive health in the next generation that don’t exposed directly to this kind of endocrine disruptors. Thus, more studies are needed to determine whether ovarian epigenome changes could alter genes that interact to govern oocyte development in the developmental programming of the germline. Besides, because the uterus can provide an environment for developing the fetus, so the change of the intrauterine estrogenic environment can affect the reprogramming of the fetus, and lead to unfavorable reproductive outcomes, for example, exposure to high levels of androgens during intrauterine life can cause changes in the methylation of some specific genes, therefore it could cause polycystic ovarian syndrome in the children [65]. Consequently, ovarian epigenome changes might affect offspring’s reproductive system function either by damage to the growing oocyte that is exposed to different metabolites, paracrine/endocrine factors, or by the changed intrauterine estrogenic environment that interacts with the embryo's genetics. Reprogramming of developing germ cells may also be affected following these exposures that have transgenerational epigenetic impacts. Reducing the methylation status in oocyte’s imprinted genes is reported following exposure to DEHP and BPA as endocrine disruptors [66]. Also, a decreased methylation of histones in cultures follicles was reported following BPA exposure [67]. Therefore, more research is needed to elucidate the epigenetic modification of female gamete following exposure to phytoestrogens.

On the other hand, we observed a marked increase in the body, ovarian weights and coefficient of the fennel-treated mice. This increase of net weight could be a marker for estrogenic properties of the various kinds of phytoestrogens in the fennel extract, although this rise was not observed in the flax-exposed mice. Our findings are consistent with previous findings supporting that genistein phytoestrogen could increase the reduced total body and ovarian weights following radiation of rats in premature ovarian failure [6]. A previous study [5] showed that exposure of post-weaning female rats to genistein could reduce the weights of the ovary, uterus, and body at a low dose while showed reversed effects in high doses.

## Conclusion

To conclude, our findings showed that pre and postnatal exposure to naturally-derived estrogens in a diet regimen supplemented with fennel or flaxseed extracts could lead to the alteration of ovarian epigenetic, as well as hormone receptor populations in the F1 adult pups. However, the full details of the underlying mechanisms of phytoestrogens' actions on the ovary have not yet been accurately defined and more studies are needed. Moreover, based on the different outcomes about the combination of these extracts compared to each extract, alone, it seems that it is still too early to introduce the safety of these plants as a portion of a diet regimen, especially during early life.

## Data Availability

The datasets used and/or analyzed during the current study are available from the corresponding author on reasonable request.

## Abbreviations

FV: *Foniculum Vulgare*
FX: Flaxseed
NMRI: Naval Medical Research Institute
PND: Post-natal day
CTL: Control
ESR: Estrogen receptor
F1: First generation
DNMT: DNA methyltransferase
HAT: Histone acetyltransferase
HDACs: Histone deacetylases
GD: Gestation day
qRT-PCR: Quantitative real time polymerase chain reaction
cDNA: Complementary DNA
SEM: Standard error of the mean
MAX: Methoxychlor
BPA: Bisphenol A
DEHP: Diethylhexyl phthalate
E2: Endogenous estradiol
AMH: Anti- Mullerian hormone
KMU: Kerman University of Medical Science
NMRI: Naval Medical Research Institute
